# Defining comprehensive biomarker‐related testing and treatment practices for advanced non‐small‐cell lung cancer: Results of a survey of U.S. oncologists

**DOI:** 10.1002/cam4.4459

**Published:** 2021-12-17

**Authors:** Kathryn F. Mileham, Caroline Schenkel, Suanna S. Bruinooge, Janet Freeman‐Daily, Upal Basu Roy, Amy Moore, Robert A. Smith, Elizabeth Garrett‐Mayer, Lauren Rosenthal, Edward B. Garon, Bruce E. Johnson, Raymond U. Osarogiagbon, Shadia Jalal, Shamsuddin Virani, Mary Weber Redman, Gerard A. Silvestri

**Affiliations:** ^1^ Levine Cancer Institute Atrium Health Charlotte North Carolina USA; ^2^ American Society of Clinical Oncology Alexandria Virginia USA; ^3^ The ROS1ders Mountain View California USA; ^4^ LUNGevity Foundation Chicago Illinois USA; ^5^ American Cancer Society National Lung Cancer Roundtable Atlanta Georgia USA; ^6^ University of California Los Angeles David Geffen School of Medicine Los Angeles California USA; ^7^ Dana‐Farber Cancer Institute Boston Massachusetts USA; ^8^ Baptist Cancer Center Memphis Tennessee USA; ^9^ Indiana University Indianapolis Indiana USA; ^10^ Aurora Health Care Burlington Wisconsin USA; ^11^ Fred Hutchinson Cancer Research Center Seattle Washington USA

**Keywords:** biomarkers, lung neoplasms, oncologists, United States

## Abstract

**Background:**

An ASCO taskforce comprised of representatives of oncology clinicians, the American Cancer Society National Lung Cancer Roundtable (NLCRT), LUNGevity, the GO_2_ Foundation for Lung Cancer, and the *ROS1*ders sought to: characterize U.S. oncologists’ biomarker ordering and treatment practices for advanced non‐small‐cell lung cancer (NSCLC); ascertain barriers to biomarker testing; and understand the impact of delays on treatment decisions.

**Methods:**

We deployed a survey to 2374 ASCO members, targeting U.S. thoracic and general oncologists.

**Results:**

We analyzed 170 eligible responses. For non‐squamous NSCLC, 97% of respondents reported ordering tests for *EGFR*, *ALK*, *ROS1*, and *BRAF*. Testing for *MET*, *RET*, and *NTRK* was reported to be higher among academic versus community providers and higher among thoracic oncologists than generalists. Most respondents considered 1 (46%) or 2 weeks (52%) an acceptable turnaround time, yet 37% usually waited three or more weeks to receive results. Respondents who waited ≥3 weeks were more likely to defer treatment until results were reviewed (63%). Community and generalist respondents who waited ≥3 weeks were more likely to initiate non‐targeted treatment while awaiting results. Respondents <5 years out of training were more likely to cite their concerns about waiting for results as a reason for not ordering biomarker testing (42%, vs. 19% with ≥6 years of experience).

**Conclusions:**

Respondents reported high biomarker testing rates in patients with NSCLC. Treatment decisions were impacted by test turnaround time and associated with practice setting and physician specialization and experience.

## Graphical abstract

U.S. oncologists who typically waited ≥3 weeks for biomarker test results were more likely to defer initiating treatment for patients with advanced non‐small‐cell lung cancer until results were reviewed. Treatment decision‐making based on biomarker test results was associated with practice setting and physician specialization and years of experience. Frequency that oncologist’s concern about delaying treatment is a reason for not testing, by years since oncology training completed.

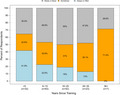

## 1 INTRODUCTION

The era of precision medicine for lung cancer commenced with accumulating data demonstrating that Epidermal Growth Factor Receptor (*EGFR*) mutations were highly predictive of response to EGFR tyrosine kinase inhibitors (TKIs). Initial testing for *EGFR* mutations started as early as 2004. However, routine testing of advanced lung adenocarcinomas was not recommended until 2011, after the IPASS and EURTAC trials were published and oral EGFR‐TKIs such as erlotinib and gefitinib were approved for treatment of patients with *EGFR* mutations.^1^, ^2^, ^3^ By mid‐2020, there was at least one FDA‐approved therapy for each of seven NSCLC oncogenic drivers (*EGFR*, *ALK*, *ROS1*, *BRAF*, *RET*, METex14, *TRK*). Additionally, multiple regimens including immune checkpoint inhibitors have received FDA approvals, some of which are based on PD‐L1 status. National Comprehensive Cancer Network (NCCN) guidelines recommend molecular testing for these actionable biomarkers, preferably as a part of broad molecular profiling including PD‐L1 testing for patients with advanced or metastatic non‐squamous NSCLC.^4^ While standard for non‐squamous NSCLC, the current recommendation for squamous cell NSCLC is to obtain PD‐L1 testing and consider molecular testing.

NCCN Guidelines advocate for broad molecular profiling as a key component in the improvement of care of patients with NSCLC and recommend completing biomarker assessment prior to therapy initiation, when clinically feasible.^4^ A retrospective analysis including more than 28,000 patients demonstrated improved outcomes—including lower risk of mortality and longer median overall survival—in patients whose tumors were tested according to NCCN guidelines.^5^ Lung cancer‐specific survival has improved by 9% in both men and women with NSCLC when comparing those diagnosed in 2001 versus 2014, partially due to biomarker testing prompting targeted treatments.^6^ Outcomes vary by biomarker‐delineated sub‐type and treatment; 5‐year survival rates for patients with metastatic NSCLC who receive targeted or immunotherapy range from 15% to 63%, versus approximately 6% for patients who receive cytotoxic chemotherapy.^1^, ^4^ Rates of comprehensive testing (i.e., for all targetable alterations), however, are not well known. In the United States, several studies have shown rising testing rates for *EGFR* and *ALK* (at or above 60% by 2016), while there are more limited data regarding testing for *BRAF* and *ROS1*.^7^, ^8^ A 2020 analysis of 150 community oncology practices found that the percentage of patients receiving biomarker testing for *EGFR*, *ALK*, and/or *ROS1* increased across practices from 2011 to 2018 (from about 55% to about 70%) but has since plateaued.^9^

Rapid advancements in the diagnostic evaluation and treatment of NSCLC challenge oncologists to adapt their practices. Therefore, an American Society of Clinical Oncology (ASCO) taskforce comprised of representatives from community and academic oncology practices, the American Cancer Society (ACS) National Lung Cancer Roundtable (NLCRT), LUNGevity, the GO_2_ Foundation for Lung Cancer, and the *ROS1*ders undertook this study to characterize U.S. oncologists’ biomarker ordering practices, to determine the types of testing platforms used, and to ascertain the barriers to testing and subsequent impact of delays in testing on treatment decisions.

## 2 METHODS

The survey was modeled after a NLCRT survey of pulmonologists.^10^ The taskforce removed or modified questions to make the survey more applicable to oncologists and added new topics specific to oncology practices. The study can be categorized as Exempt Research under the 2018 Common Rule because the information obtained through the survey procedures could not be readily linked to the human subjects and did not place the subjects at any risk of liability or damage to standing, advancement, reputation, etc.^11^ Therefore, the survey instrument was not submitted for review by an Institutional Review Board (IRB). However, the survey was reviewed by ASCO’s Research Services Committee, which considers design and ethical issues.

The survey was sent to a sample of 2374 active physician, nurse practitioner (NP), and physician assistant (PA) ASCO members in the United States that was comprised of 25% lung specialists and 75% generalists. Due to a low initial response rate, the survey was sent to additional ASCO members and non‐members through taskforce networks, which eliminated the ability to track recipient distribution and thus final response rate. The survey was available from July 7, 2020 through September 24, 2020. Because the survey was open during the COVID‐19 pandemic, respondents were asked to report on their pre‐pandemic patient load and clinical behavior. The goal was to collect at least 400 responses to ensure adequate precision, estimates, and ability to compare subgroups of respondents. However, we assumed that keeping the survey open more than 3 months would not yield many additional respondents. We summarized results using proportions, made comparisons across groups using Fisher's exact test, and used a two‐sided alpha level of 0.05 to determine statistical significance. The survey instrument is included in the Appendix.

## 3 RESULTS

Of 194 complete, unique responses, 170 from oncology physicians (general and thoracic), NPs, and PAs were eligible for analysis. Responses from other oncology subspecialties (e.g., radiation [*n* = 8], and pediatrics [*n* = 2]) were excluded. Ninety‐eight percent of respondents were physicians.

Respondents’ characteristics are presented in Table 1. Respondents were evenly split between low lung cancer specialization/volume (i.e., general oncologists who saw ≤50% patients with lung cancer in an average month) and high lung cancer specialization/volume (i.e., thoracic oncologists or those with ≥51% patients with lung cancer per month). Nearly 60% of respondents practiced primarily at an academic site (defined by the presence of a fellowship program).

**TABLE 1:** Survey respondent characteristics (*N* = 170)

	*n* (%)
Lung specialization	
General hematologist/oncologist with LOW percentage (1%–50%) of lung cancer patients in average monthly caseload (includes 3 NPs/PAs)	87 (51.5)
General hematologist/oncologist with HIGH percentage (51%−100%) of lung cancer patients in average monthly caseload OR Thoracic oncologist	82 (48.5)
Academic practice (defined as practice/institution conducts a fellowship program)	
Yes	101 (59.4)
No	69 (40.6)
Hospital affiliation(s)	
General hospital (only)	42 (24.7)
Tertiary care hospital (only)	29 (17.1)
NCI‐designated cancer center (only)	21 (12.4)
Academic cancer center (only)	16 (9.4)
VA/military hospital (only)	6 (3.5)
NCI designation	
NCI‐designated cancer center (only or in combination with any other)	60 (35.3)
Not NCI‐designated cancer center	110 (64.7)
Practice region	
South	55 (33.1)
Midwest	48 (28.9)
Northeast	38 (22.9)
West	25 (16.1)
Years since completing training	
<5	32 (19.2)
6–15	63 (37.7)
16–25	42 (25.1)
26+	30 (18.0)
Total new cancer cases per month	
1–19	102 (59.9)
20–29	42 (24.7)
30+	26 (15.3)
Access to services	
General surgery and/or surgical oncology	
Within practice/institution	157 (92.4)
Through affiliation with another practice/institution	13 (7.6)
Not available	0 (0.0)
Radiation oncology	
Within practice/institution	151 (88.8)
Through affiliation with another practice/institution	18 (10.6)
Not available	0 (0.0)
Pulmonology	
Within practice/institution	149 (87.6)
Through affiliation with another practice/institution	20 (11.8)
Not available	1 (0.6)
Patient Navigators	
Within practice/institution	147 (86.5)
Through affiliation with another practice/institution	6 (3.5)
Not available	17 (10.0)
Multidisciplinary tumor board	
Within practice/institution	149 (87.6)
Through affiliation with another practice/institution	18 (10.6)
Not available	2 (1.2)
Molecular tumor board	
Within practice/institution	95 (55.9)
Through affiliation with another practice/institution	47 (27.6)
Not available	27 (15.9)
Molecular pathology	
Within practice/institution	103 (60.6)
Through affiliation with another practice/institution	51 (30)
Not available	16 (9.4)
Average wait time for biomarker testing results	
Within 1 week	8 (4.7)
Within 2 weeks	99 (58.2)
Within 3 weeks	48 (28.2)
Within 4 weeks	14 (8.2)
Acceptable wait time for biomarker testing results	
Within 1 week	78 (45.9)
Within 2 weeks	89 (52.4)
Within 3 weeks	2 (1.2)
Within 4 weeks	1 (0.6)
If it takes longer than 2 weeks to receive results for genomic biomarker testing, do you usually:	
Continue to defer treatment until all biomarker results are available and reviewed	87 (52.4)
Initiate a non‐targeted systemic treatment before all biomarker results are available and reviewed	79 (47.6)

### 3.1 Specimen acquisition and testing methods

Most respondents (68%) reported that they were responsible for ordering biomarker testing for their patients (vs. a pulmonologist or pathologist), while 21% reported that testing occurred reflexively/automatically. The most common method (47%) for obtaining samples for biomarker testing was endobronchial ultrasound‐guided transbronchial needle aspiration (EBUS‐TBNA), followed by percutaneous biopsy by interventional radiologist (31%). Generalists were less likely to report EBUS‐TBNA as their most common method compared to thoracic oncologists (37% vs. 58%) and more likely to use percutaneous biopsy as their most common method than thoracic oncologists (39% vs. 24%) (*p *= 0.017). Whereas only 9% of respondents from community sites obtained in‐house biomarker testing more than three‐quarters of the time, 28% from academic sites utilized in‐house testing with this frequency (*p *= 0.003). Exclusive use of commercial labs was more common among respondents from community sites (54%, vs. 21% from academic sites, *p *< 0.001).

Ninety‐three percent of respondents reported utilizing multi‐gene panels more frequently than single gene panels. Testing for biomarkers associated with the earliest FDA‐approved agents (2013–2017) for non‐squamous NSCLC (*EGFR*, *ALK*, *ROS1*, and *BRAF*) were reported by 97% of respondents, with no difference between community versus academic oncologists, or generalists versus specialists. Testing for biomarkers associated with agents approved starting in 2018 for non‐squamous NSCLC was higher among providers at academic sites than those at community practices (*MET*: 95% vs. 81% [*p *= 0.005]; *RET*: 91% versus 80% [*p *= 0.041]; NTRK: 89% vs. 81% [*p *= 0.18]) and higher among lung cancer specialists than generalists (*MET*: 95% vs. 84% [*p *= 0.024]; *RET*: 93% versus 82% [*p *= 0.040]; NTRK: 93% versus 79% [*p *= 0.015]). Testing for *KRAS* and HER2 biomarkers, neither of which had FDA‐approved targeted therapies at survey time, was above 70% for all subsets and above 85% among thoracic oncologists and providers at academic sites.

Reported biomarker testing rates were also high in patients with squamous NSCLC. The frequency of testing for *EGFR*, *BRAF*, *NTRK*, *MET*, *RET*, *KRAS*, *ROS1*, *ALK*, and HER2 was between 69% and 80% overall, with no significant difference between provider subgroups.

Ninety‐five percent of respondents reported testing for both actionable biomarkers and PD‐L1 in non‐squamous NSCLC patients prior to starting immunotherapy. If a patient with NSCLC was started on a treatment regimen based on PD‐L1 expression and an actionable mutation was later identified, two‐thirds of respondents would change treatment to corresponding targeted therapy, while one‐third would either not change treatment or would change only if initial treatment was intolerable or led to progression. Additionally, if an actionable mutation was identified from circulating‐tumor DNA and no PD‐L1 expression was available from tissue, 30% of specialists reported “never obtaining a fresh tissue biopsy, even when possible” compared with only 8% of generalists (*p *= 0.001).

Rates of blood‐based molecular testing were low, and no significant differences in use were observed between community and academic respondents or between generalists and specialists. In non‐squamous NSCLC, the largest percentage of respondents (45%) reported ordering blood‐based molecular testing for less than 25% of patients. In squamous NSCLC, low rates of blood‐based molecular testing were more common, with 68% of respondents ordering for <25% of patients and 22% of respondents not ordering blood testing at all. When considering all cases of NSCLC regardless of histology, 59% of respondents reported having used blood‐based molecular testing alone (without tissue) to make initial treatment decisions; 77% of respondents reported having used blood‐only testing to make treatment decisions at progression.

### 3.2 Testing patterns, barriers, and strategies for improvement

All survey respondents reported high rates of biomarker testing for patients with non‐squamous NSCLC. Of respondents testing more than three quarters of patients with non‐squamous NSCLC, 92% tested at diagnosis and 85% tested at progression.

There were differences in biomarker testing for patients with squamous NSCLC. Generalists were more likely to report ordering biomarker testing for more than 75% of their patients with squamous NSCLC at diagnosis (67%, compared to 30% of specialists [*p *< 0.001]). There was a smaller gap between respondents from community and academic sites regarding testing squamous NSCLC at diagnosis: 57% at community sites reported testing more than three‐quarters of these patients, compared to 44% at academic sites (*p *= 0.12). Oncologists within five years of training were more likely to report high levels of testing (>75% of patients) at progression and diagnosis and for both squamous and non‐squamous disease. Most respondents reported that less than 20% of their patients with advanced NSCLC did not proceed with further treatment, with comparable percentages between generalists versus specialists (85% vs. 82%) and community versus academic sites (82% vs. 84%).

### 3.3 Barriers to biomarker testing

**FIGURE 1:**
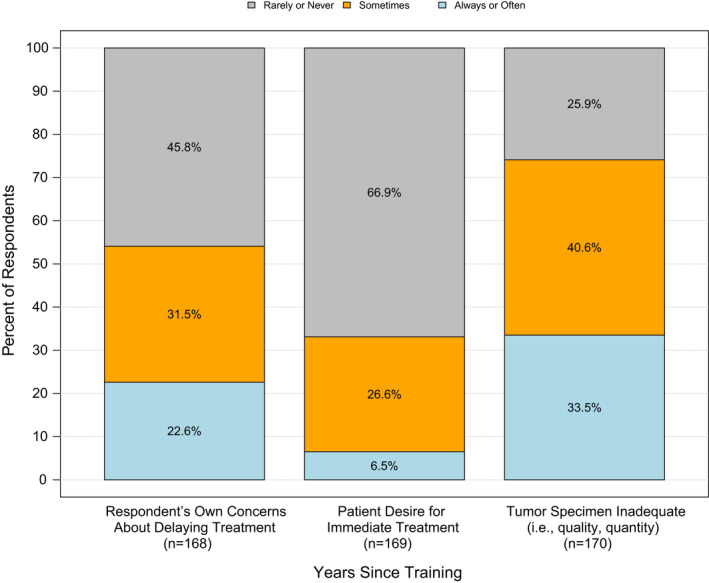
Most frequent reasons for not ordering biomarker testing

Respondents were asked to select top reasons why biomarker testing did not occur (Figure 1). “Inadequate tumor specimens” was most frequently selected (34%) as “always or often” a barrier. Twenty‐three percent of respondents stated that their concern about delaying treatment while waiting for test results was “always or often” a reason for not ordering biomarker testing, and half reported typically initiating a non‐targeted treatment if biomarker test results were not available after 2 weeks.

**FIGURE 2:**
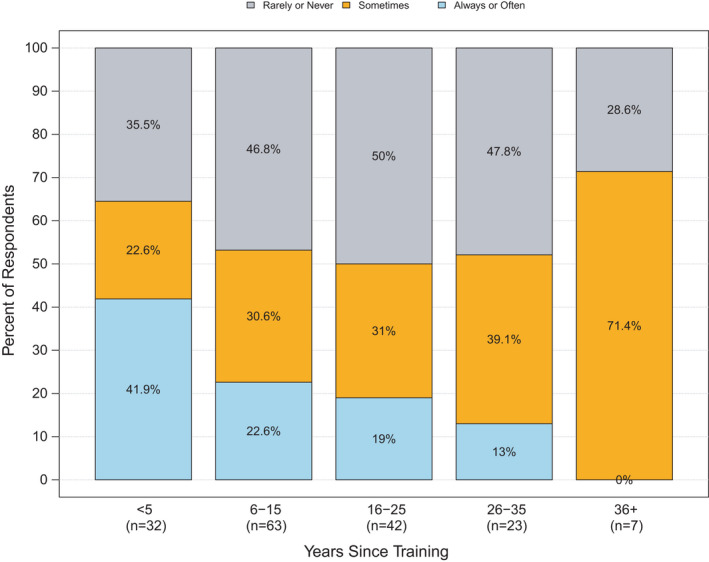
Frequency that oncologist's concern about delaying treatment is a reason for not testing, by years since oncology training completed

Providers farther out from training were less likely to report concern about waiting for test results as “always or often” a reason for not ordering biomarker testing, compared to providers who recently completed oncology training (Figure 2). Forty‐two percent of oncologists who were within 5 years of training reported that their concern about waiting for biomarker test results was a frequent reason for not ordering testing compared to 18% of those ≥6 years out of training (*p *= 0.025).

The majority (71%) of respondents reported that their practices had implemented strategies to increase biomarker testing among patients with NSCLC, with comparable percentages (73% vs. 67%) between academic and community sites. Coordination across multidisciplinary treatment teams was the most common strategy provided, occurring at 85% of these respondents’ practices. This type of coordination was slightly more common at academic (89%) versus community sites (78%). Clinician education was the second‐most common strategy to increase biomarker testing and was equally common at academic (68%) and community practices (70%). Of respondents from practices with specific improvement strategies, 60% reported that testing rates had improved.

### 3.4 Time to receipt of test results and impact on treatment decision making

There was a discrepancy between what oncologists considered an acceptable wait time for biomarker test results and how long they typically waited for results. Most considered 1 (46%) or 2 weeks (52%) an acceptable turnaround time, yet 37% reported usually waiting ≥3 weeks to receive test results.

When asked about treatment decisions if biomarker testing results were not received within 2 weeks, the majority of respondents (52%) reported that they would typically defer treatment until review of test results, rather than initiate non‐targeted treatment (48%). Respondents who usually waited ≥3 weeks to receive biomarker test results were more likely to defer treatment until results were reviewed (63%). Respondents who usually waited 1–2 weeks were significantly less likely to report that they would typically defer treatment (46%; *p *= 0.038). Examining the same data about actions taken when results were not received within 2 weeks by practice type and specialization also revealed significantly different clinical actions. Respondents from community practices were more likely to report typically initiating non‐targeted treatment than respondents from academic practices (59% vs. 40%; *p *= 0.013). General oncologists were more likely to initiate non‐targeted systemic treatment than specialists (55% vs. 39%; *p *= 0.045).

## 4 DISCUSSION

This survey provides key insights into the current landscape of biomarker testing. First, respondents reported high biomarker testing rates that match or exceed levels observed by recent U.S.‐based studies, whose results vary significantly by year, practice setting, and biomarker.^7^, ^9^, ^12^ Rates of guideline utilization (NCCN, ASCO, or other) among respondents, which correlated with high testing rates and use of multi‐gene panels, were high overall (98.8%) and equal among academic versus community respondents and generalist versus specialist respondents. Consistent testing for *EGFR*, *ALK*, and *ROS1* for patients with non‐squamous NSCLC reported by 100% of respondents (98% for *BRAF*) suggests familiarity with earlier approved treatments associated with those biomarkers regardless of practice setting, specialization, or other respondent characteristics.

These findings are similar to those from a retrospective analysis of data collected in 2017 from seven cancer programs, three of which were community‐based, although direct comparisons of our self‐reported survey results with these real‐world data analyses have limitations.^12^ Mason et al reported overall testing rates in advanced non‐squamous NSCLC for *EGFR*, *ALK*, and *ROS1* from 85% to 100%, with rates of 100% across these mutations in the academic setting. The testing rate for *ROS1* in the community setting was 85% (*p *= 0.0015). During the years preceding the Mason et al analysis, rates of testing for *EGFR*, *ALK*, and *ROS1* were lower (25%–70%).^7^, ^13^, ^14^ The number of mutations tested was lower, the use of large multi‐gene panels was limited, and testing costs were likely higher, all which may have limited testing. Our survey addressed testing rates by NSCLC histology, mutations with more recently approved targeted therapy (*TRK*, *RET*, *MET*), plus those with therapy under approval consideration (*KRAS*, HER2) at the time of survey conduction.

Reported rates of testing for advanced non‐squamous NSCLC at diagnosis meet guideline standards. However, such a high rate of guideline adherence seems improbable. The overall reported rate of comprehensive biomarker testing for advanced squamous NSCLC at diagnosis was surprisingly high given that actionable mutations are less frequently identified in this subtype and guidelines for biomarker testing are not as emphatic with squamous as non‐squamous disease. This survey identified differences in biomarker testing of squamous NSCLC based on specialization. High rates of testing by general oncologists may indicate their challenges in keeping pace with current recommendations, resulting in an inappropriate blanket testing approach, rather than a selective approach relying on guidelines and recent data within a rapidly evolving landscape.

Second, these survey findings suggest that clinical decision‐making for NSCLC is influenced by the range of wait times for biomarker test results as well as by the oncologist's professional and practice characteristics. Since this survey defined waiting time as ordering to receipt of results, delays could occur at any point along the timeline including biopsy (e.g., if additional tissue or blood is needed), pathology send out, actual testing, and analysis. This survey also found that more experienced clinicians (especially those with ≥51% of patients with lung cancer and/or those later in their career) were more likely to defer treatment with non‐targeted therapies while waiting for results. When asked about factors that impede but do not necessarily prevent genomic biomarker testing, respondents were more likely to cite their own concerns as “always” or “often” a barrier than their patients’ concerns.

More extensive education about the patient‐centered benefit of waiting for biomarker results prior to initiating treatment may be warranted. NCCN guidelines advocate that clinicians should obtain molecular test results for actionable biomarkers prior to administering first‐line therapy, when feasible.^4^ Metastatic NSCLC is variably responsive to treatment based on the oncogenic driver. For example, a patient with metastatic NSCLC with PD‐L1 expression levels ≥1% and a targetable driver oncogene is more appropriately treated with first‐line oncogene‐driven therapy rather than immunotherapy‐based treatment. Targeted therapies are better tolerated and yield higher response rates than immunotherapy‐based regimens.^15^, ^16^, ^17^ Moreover, targeted therapy after immunotherapy has been associated with increased treatment‐related toxicities.^18^, ^19^ Patients may also be ineligible for first‐line targeted treatment trials if chemotherapy has already been initiated.

The use of clinical pathways is associated with guideline‐adherent biomarker testing and targeted therapy for NSCLC in both academic and community practice settings.^12^ While nearly all survey respondents reported following one or more guidelines for treatment selection, fewer than 60% from either academic or community practices indicated that the use of clinical guidelines or pathways was a part of their practice's strategy to increase biomarker testing for NSCLC. This is paradoxical and speaks to the need to target interventions to clinicians in community practices, oncologists who treat fewer patients with lung cancer, and early career providers to convey the importance of guideline adherence and the value of waiting for biomarker results before initiating treatment.

Finally, strategies should be employed to decrease time to receipt of results. This could include coordinated efforts between pathologists and tissue procurers to standardize processes, such as reflexive testing.^20^ Companies providing biomarker testing should recognize that test turnaround time influences treatment initiation decisions. All efforts to achieve the desired 1‐ to 2‐week turnaround interval should be implemented.

We acknowledge that generalizability of these survey findings is limited by the low response rate and respondent selection bias. Nearly all responses (95%) were received via outreach through ASCO channels, which included ASCO volunteer committees. The proportion of lung specialists responding to the survey (49%) was higher than the proportion included in the original survey sample (25%), likely due to specialists’ interest in the topic. Most respondents reported access to molecular tumor boards and molecular pathology resources, suggesting knowledge of or access to resources regarding biomarker testing that does not reflect the experience of all U.S. oncologists. Additionally, this analysis is based on self‐report, which is susceptible to recall bias and overestimation of guideline compliance. The level of biomarker testing may be much higher than true practice. Despite these limitations, this analysis adds valuable data to the literature. Our findings suggest that certain subsets of oncologists who treat advanced NSCLC—such as community and early career physicians—should be targeted for educational intervention regarding the appropriate selection and timing of therapies for patients awaiting biomarker test results.

The taskforce is also conducting an analysis of electronic health record‐based data (from ASCO’s CancerLinQ Discovery dataset) to determine biomarker testing patterns among a cohort of patients with advanced NSCLC. That analysis will allow for qualitative comparison between real‐world evidence and the self‐reported data collected in this survey.

## 5 CONCLUSION

Despite oncologists’ high self‐reported biomarker testing rates for NSCLC, treatment decisions often hinge on the time interval of receiving test results—specifically 2 weeks—rather than the results themselves.

## CONFLICT OF INTEREST

The authors of this work disclose the following financial relationships with for‐profit healthcare companies: [Fam = immediate family member; Inst = institution received].

Basu Roy: Honoraria: AstraZeneca (Inst); Research Funding: Merck (Inst), Boehringer Ingelheim (Inst), BluePrint Medicine (Inst), Genentech (Inst), Bristol‐Myers Squibb (Inst), G1 Therapeutics (Inst), Janssen Oncology (Inst). Bruinooge: No relationships to disclose. Freeman‐Daily: Honoraria: AstraZeneca; Consulting or Advisory Role: Genentech/Roche; Travel, Accommodations, Expenses: Questex LLC, Turning Point Therapeutics. Garon: Consulting or Advisory Role: Dracen, EMD Serono, Novarti, GlaxoSmithKline, Merck, Boehringer Ingleheim, Shionogi, Eisai, Bristol‐Myers Squibb, ABL Bio, Sanofi, Xilio; Research Funding: Merck (Inst), Genentech (Inst), AstraZeneca (Inst), Novartis (Inst), Lilly (Inst), Bristol‐Myers Squibb (Inst), Mirati Therapeutics (Inst), Dynavax (Inst), Iovance Biotherapeutics (Inst), Neon Therapeutics (Inst), EMD Serono (Inst). Garrett‐Mayer: Consulting or Advisory Role: Deciphera, TYME. Jalal: Consulting or Advisory Role: Adaptimmune; Research Funding: AstraZeneca/MedImmune, Tesaro, Astex Pharmaceuticals. Johnson: Consulting or Advisory Role: Novartis, Foundation Medicine, Hengrui Therapeutics, Daiich Sankyo, Chugai Pharmaceuticals, Lilly, Checkpoint Therapeutics, G1 Therapeutics, Jazz Pharmaceuticals, GlaxoSmithKline, Boston Pharmaceuticals, Janssen Scientific Affairs, Genentech, Hengrui USA Ltd; Research Funding: Novartis, Novartis (Inst); Patents, Royalties, Other Intellectual Property: Dana‐Farber Cancer Institute. Mileham: Honoraria: Takeda, Bristol‐Myers Squibb; Consulting or Advisory Role: AstraZeneca, Bayer; Speakers’ Bureau: Merck; Research Funding: Celgene. Moore: Consulting or Advisory Role: Guardant Health. Osarogiagbon: Stock and Other Ownership Interests: Lilly, Pfizer, Gilead; Honoraria: Biodes; Consulting or Advisory Role: Association of Community Cancer Centers, AstraZeneca, American Cancer Society, Triptych Health Partners; Other Relationship: Oncobox Device, Inc. Redman: Consulting or Advisory Role: AstraZeneca, Merck. Rosenthal: No relationships to disclose. Schenkel: No relationships to disclose. Silvestri: Consulting or Advisory Role: Biodesix inc, Olympus America, AstraZeneca; Research Funding: NIH, NCI, Biodesix in, SEER, Nucleix. Smith: Other relationship: Dr. Smith is employed by the American Cancer Society, which receives grants from private companies and corporate foundations, in the health sector, outside of the submitted work. His salary is solely funded through American Cancer Society funds. Virani: Employment: Walgreens (Fam); Stock and Other Ownership Interests: BMY, WBA.

## AUTHOR CONTRIBUTIONS

Kathryn F. Mileham: conceptualization, methodology, project administration, supervision, validation, visualization, writing – original draft, writing – review and editing. Caroline Schenkel: conceptualization, data curation, formal analysis, investigation, methodology, project administration, resources, supervision, visualization, writing – original draft, writing – review and editing. Suanna S. Bruinooge: conceptualization, methodology, project administration, resources, supervision, visualization, writing – original draft, writing – review and editing. Janet Freeman‐Daily: conceptualization, methodology, supervision, writing – review and editing. Upal Basu Roy, PhD: conceptualization, methodology, supervision, writing – review and editing. Amy Moore: conceptualization, methodology, resources, supervision, writing – review and editing. Robert A. Smith: conceptualization, methodology, supervision, writing – review and editing. Elizabeth Garrett‐Mayer: conceptualization, data curation, formal analysis, methodology, supervision, visualization, writing – original draft, writing – review and editing. Lauren Rosenthal: conceptualization, methodology, supervision, writing – review and editing. Edward B. Garon: conceptualization, methodology, supervision, writing – review and editing. Bruce E. Johnson: conceptualization, methodology, supervision, writing – review and editing. Raymond U. Osarogiagbon: conceptualization, methodology, supervision, writing – review and editing. Shadia Jalal, MD: conceptualization, methodology, supervision, writing – review and editing. Shamsuddin Virani: conceptualization, methodology, supervision, writing – review and editing. Mary Weber Redman: conceptualization, methodology, supervision, writing – review and editing. Gerard A. Silvestri: conceptualization, methodology, supervision, validation, writing – original draft, writing – review and editing.

## Supporting information

Supplementary Material

## Data Availability

The data that support the findings of this study are available from the corresponding author upon reasonable request.
