# Supplementary material for: Defining comprehensive biomarker‐related testing and treatment practices for advanced non‐small‐cell lung cancer: Results of a survey of U.S. oncologists
Source: Cancer Med. 2021 Dec 17;11(2):530–8. doi: 10.1002/cam4.4459 (PMC8729042; doi:10.1002/cam4.4459)
Supplement: Supplementary file 1 — Supplementary Material [file CAM4-11-530-s001.pdf]

Understanding Comprehensive Biomarker-Related Testing and Treatment Practices for Lung Cancer in the Oncologist Community

Dear ASCO member,

The American Society of Clinical Oncology, American Cancer Society, ROS1ders, LUNGevity, GO2 Foundation for Lung Cancer, and other member organizations of the National Lung Cancer Roundtable (NLCRT) are conducting a foundational study with oncologists to understand current practice for biomarker testing and treatment for patients with advanced stage (stage 3b, 3c, or 4) squamous and non-squamous non-small cell lung cancer (NSCLC). We are interested in testing rates, situations in which testing occurs, and barriers to testing and biomarker-related treatments. We intend to publish the results of this research in order to advance the deployment of these practices for the benefit of patients. This survey will take approximately 15-17 minutes to complete. We hope you will participate so, together, we can identify opportunities to support the diagnostic and treatment planning.

Please note that we are asking about your experience with biomarker testing PRIOR TO COVID-19. Regardless of your frequency of biomarker use, please provide your answers in the context of cancer care PRIOR TO COVID-19.

We will keep your data confidential and nonidentifiable. We will only analyze deidentified data and publish aggregate results. Following publication of our initial manuscript, we plan to make deidentified data available to external researchers who submit a valid research proposal to ASCO’s Data Library.

Thank you for consenting to participate in the survey and for your time, effort, and consideration. We appreciate your intellectual contribution.

Best regards,

Kathryn F. Mileham, MD, FACP

Levine Cancer Institute

American Society of Clinical Oncology

Gerard Silvestri, MD, MS, FCCP

Medical University of South Carolina

National Lung Cancer Roundtable

Bruce E. Johnson, MD, FASCO

Dana-Farber Cancer Institute

National Lung Cancer Roundtable

Robert A. Smith, PhD

American Cancer Society

National Lung Cancer Roundtable

Janet Freeman-Daily, MS, Eng

ROS1ders

Upal Basu Roy, PhD, MPH

LUNGevity Foundation

Amy Moore, PhD

GO2 Foundation for Lung Cancer

YOUR DEMOGRAPHIC INFORMATION

Please enter your email address.<br><br>(Your responses will be kept anonymous. Collecting your email ensures that we do not follow-up with you to respond to the survey after you have already done so.)	________________________
Please enter your ASCO ID number.	________________________
Is your primary practice location in the United States or a U.S. territory?	◯ Yes<br>◯ No

1. Are you a ...

- ◯ General Hematologist/Oncologist
- ◯ Thoracic Oncologist
- ◯ Nurse Practitioner or PA
- ◯ Surgeon
- ◯ Surgical Oncologist
- ◯ Gynecologic Oncologist
- ◯ Radiation Oncologist
- ◯ Pediatric Oncologist
- ◯ Pathologist

1b. Do you provide care for at least 1 new lung cancer patient in a typical month?

- ◯ Yes
- ◯ No

2. Please indicate the number of years since you finished training.

- ◯ < 5
- ◯ 6-15
- ◯ 16-25
- ◯ 26-35
- ◯ 36+

3. Which of the following best describes your practice setting?

- ◯ Physician-owned practice or group (including multi-site network)
- ◯ Hospital or health system-owned practice, group, or outpatient department (including state-funded institutions)
- ◯ Government (Federal: [Public Health System, Military, VA, etc.] or State)
- ◯ Industry (do not work in a practice setting)
- ◯ Other

3b. Please describe:

____

4. Does your practice engage in the following activities? [check all that apply]

- ☐ Conducts a fellowship program
- ☐ Hosts visiting fellows for rotations from an academic institution
- ☐ Research

5. Please enter the 5-digit zip code of the PRIMARY practice location at which YOU treat patients.

____

6. Which of the following best describes your primary hospital affiliation? [check all that apply]

- ☐ NCI-designated cancer center
- ☐ Tertiary care hospital
- ☐ Academic cancer center
- ☐ General hospital
- ☐ VA or military hospital
- ☐ Other

6b. Please describe:

____

7. Please indicate which of the following medical specialties/services are available EITHER within your practice or institution OR through affiliation/agreement with other practices/institutions. [check all that apply]

	WITHIN my Practice/Institution	THROUGH AFFILIATION/AGREEMENT with other Practice(s)/Institution(s)	NOT Available
General Surgery and/or Surgical Oncology	◯	◯	◯
Radiation Oncology	◯	◯	◯
Pulmonology	◯	◯	◯
Molecular Pathology (specifically genomic molecular testing)	◯	◯	◯
Multidisciplinary Tumor Board (with thoracic oncology expertise)	◯	◯	◯
Molecular Tumor Board (with molecular pathology expertise)	◯	◯	◯
Patient Navigators (whether nurses or trained patients)	◯	◯	◯

NOTE: The rest of the survey asks about your recent experience PRIOR to the COVID-19 pandemic.

PERSONAL CLINICAL EXPERIENCE

8. On average, what is the total number of new cancer cases (across all cancers you treat) that you see in a typical month? __________________________________

9. What percentage of your new cancer cases in a typical month are lung cancer?
   - ◯ 0
   - ◯ 1% - 25%
   - ◯ 26% - 50%
   - ◯ 51% - 75%
   - ◯ 76% - 99%
   - ◯ 100%

10. Within your lung cancer cases, what is the average percentage with advanced stage (stage 3b, 3c, or 4) non-small cell lung cancer (NSCLC)?
    - ◯ 1% - 25%
    - ◯ 26% - 50%
    - ◯ 51% - 75%
    - ◯ 76% - 99%
    - ◯ 100%

11. What is the average percentage of your advanced NSCLC patients who either 1) are not candidates for systemic therapy or 2) do NOT desire further treatment (including clinical trials)? __________________________________

NOTE: The rest of the survey asks about two groups of your patients with advanced stage (stage 3b, 3c, or 4) non-small cell lung cancer (aNSCLC) - 1) nonsquamous cell and 2) squamous cell. Please think specifically about those patients when answering the remaining survey questions.

INSTITUTIONAL SAMPLING PRACTICES

12. Please rank the top three most frequently used techniques (by your own order or others' determination) among patients you treat to obtain a specimen for pathologic diagnosis of aNSCLC. If you are unsure of rankings, select 'unsure how to rank' for 1, 2 or 3.

	Endobronchial ultrasound-guided transbronchial needle aspiration (EBUS-TBNA)	Transthoracic needle biopsy	Surgical specimen	Mediastin-oscopy	Thora-centesis	Liquid biopsy	Percuta-neous biopsy (via IR)	Unsure how to rank
1	☐	☐	☐	☐	☐	☐	☐	☐
2	☐	☐	☐	☐	☐	☐	☐	☐
3	☐	☐	☐	☐	☐	☐	☐	☐

13. Why do you think your first-ranked technique is used (by your choice or others' choice)? [check all that apply]

☐ Ease of access to the technology
☐ Better tissue sampling for testing
☐ Speed in scheduling so patient doesn't have to wait
☐ Anatomic location of the tumor specimen
☐ Resources are readily available for this procedure
☐ Does not require referring patient to a different facility
☐ Not sure
☐ None of the above

NOTE: For the purposes of this survey, genomic biomarker testing is defined as single-gene or multi-gene panel (such as next-generation sequencing) testing for genomic markers (ALK, EGFR, ROS-1, KRAS, BRAF, NTRK, etc.) – excluding immune biomarker testing.

GENOMIC BIOMARKER TESTING ON TISSUE SAMPLES (NOTE: IMMUNE BIOMARKER TESTING IN FOLLOWING SECTION)

14. At your practice/institution who most often ORDERS biomarker testing for patients with aNSCLC?

◯ I order it
◯ Pulmonologist orders it
◯ Pathologist orders it
◯ Surgeon/surgical oncologist orders it
◯ Testing is completed reflexively/automatically
◯ Not sure

15. Think about your NSCLC patients who have recurred or progressed to advanced stage (3b, 3c, and 4). For what percentage of these patients do you request a fresh tissue BIOPSY for GENOMIC BIOMARKER TESTING?

	0%	1% - 25%	26% - 50%	51% - 75%	76% - 99%	100%
Nonsquamous aNSCLC Patient %	◯	◯	◯	◯	◯	◯
Squamous aNSCLC Patient %	◯	◯	◯	◯	◯	◯

16. What is the average turnaround time it takes from ordering to receipt of genomic biomarker testing?

◯ Within 1 week
◯ Within 2 weeks
◯ Within 3 weeks
◯ Within 4 weeks
◯ >4 weeks

17. In your opinion, what is an acceptable turnaround time from order to receipt of results for genomic biomarker testing?

◯ Within 1 week
◯ Within 2 weeks
◯ Within 3 weeks
◯ Within 4 weeks
◯ >4 weeks

18. If it takes longer than 2 weeks to receive results for genomic biomarker testing, do you usually:

◯ Continue to defer treatment until all biomarker results are available and reviewed
◯ Initiate a non-targeted systemic treatment before all biomarker results are available and reviewed

19. What percentage of your patients with aNSCLC at diagnosis receive genomic biomarker testing?

	0%	1% - 25%	26% - 50%	51% - 75%	76% - 99%	100%
Nonsquamous aNSCLC Patient %	◯	◯	◯	◯	◯	◯
Squamous aNSCLC Patient %	◯	◯	◯	◯	◯	◯

20. Consider your patients who have progressed to aNSCLC and who are interested in learning about additional treatment options. For what percentage of these patients do you order genomic biomarker testing?

	0%	1% - 25%	26% - 50%	51% - 75%	76% - 99%	100%
Nonsquamous aNSCLC Patient %	◯	◯	◯	◯	◯	◯
Squamous aNSCLC Patient %	◯	◯	◯	◯	◯	◯

21. For patients with nonsquamous aNSCLC, what is your most frequent platform used for genomic biomarker testing?

◯ Single gene (whether sequentially or concurrently)
◯ Multi-gene panel (e.g., next generation sequencing)

22. Which genes do you routinely order testing for your patients with nonsquamous aNSCLC (either as single gene or as part of a panel)?

	Yes	No
EGFR	◯	◯
ALK	◯	◯
BRAF	◯	◯
ROS1	◯	◯
NTRK	◯	◯
HER2	◯	◯
KRAS	◯	◯

MET	◯	◯
RET	◯	◯

23. For patients with squamous aNSCLC, what is your most frequent platform used for genomic biomarker testing?
    - ◯ Single gene (whether sequentially or concurrently)
    - ◯ Multi-gene panel (e.g., next generation sequencing)

24. Which genes do you routinely order testing for your patients with squamous aNSCLC (either as single gene or as part of a panel)?

	Yes	No
EGFR	◯	◯
ALK	◯	◯
BRAF	◯	◯
ROS1	◯	◯
NTRK	◯	◯
HER2	◯	◯
KRAS	◯	◯
MET	◯	◯
RET	◯	◯

25. For what percentage of aNSCLC samples does your practice/institution use the following type of lab for genomic biomarker testing?

In-house lab — 0 to 100

(Place a mark on the scale above)

Lab within a different hospital or health system — 0 to 100

(Place a mark on the scale above)

Commercial lab (NOT within a hospital or health system - e.g., Foundation Medicine, Caris, Tempus, etc.) — 0 to 100

(Place a mark on the scale above)

Total (adding up to 100%) ______________________

GENOMIC BIOMARKER TESTING ON BLOOD SAMPLES

26. For what percentage of your patients with aNSCLC do you conduct an analysis of blood for circulating tumor DNA?

	0%	1% - 25%	26% - 50%	51% - 75%	75% - 99%	100%
Nonsquamous aNSCLC Patient %	◯	◯	◯	◯	◯	◯
Squamous aNSCLC Patient %	◯	◯	◯	◯	◯	◯

27. Do you ever use a liquid blood draw (liquid biopsy/plasma-based biopsy) alone to make initial treatment decisions about targeted therapies for aNSCLC patients?

- ◯ Yes
- ◯ No
- ◯ I do not know

28. Do you ever use a liquid blood draw (liquid biopsy/plasma-based biopsy) alone to make treatment decisions about targeted therapies at recurrence or progression for aNSCLC patients?

- ◯ Yes
- ◯ No
- ◯ I do not know

29. For frontline therapy for nonsquamous aNSCLC cases where circulating tumor DNA analysis shows no alterations AND you have insufficient tissue for biomarker testing, do you obtain a fresh biopsy?

- ◯ Almost always obtain a fresh tissue biopsy, where possible
- ◯ Usually obtain a fresh tissue biopsy, where possible
- ◯ Sometimes obtain a fresh tissue biopsy, where possible
- ◯ Never obtain a fresh tissue biopsy, even if possible
- ◯ Wait until the patient progresses to obtain a fresh biopsy

30. If you obtain evidence of an actionable genomic biomarker in liquid biopsy test results for a patient with nonsquamous or squamous aNSCLC, but have no PD-L1 expression level from biomarker testing with tissue, which choice best describes your subsequent approach?

- ◯ Always obtain a fresh tissue biopsy (if possible) to evaluate PD-L1 expression
- ◯ Sometimes obtain a fresh tissue biopsy (if possible) to evaluate PD-L1 expression
- ◯ Sometimes obtain a fresh tissue biopsy (if possible) to evaluate for PD-L1 expression unless an actionable alteration has been identified
- ◯ Never get a fresh tissue biopsy (even where possible) to evaluate for PD-L1 expression

IMMUNE BIOMARKER TESTING ON TISSUE SAMPLES

NOTE: The following question asks about testing for immunotherapy treatments for patients with aNSCLC (BOTH nonsquamous and squamous).

31. Consider your patients who have progressed to aNSCLC and who are interested in learning about additional treatment options. For what percentage of these patients do you order testing for immunotherapy?

	0%	1% - 25%	26% - 50%	51% - 75%	76% - 99%	100%
Nonsquamous aNSCLC Patient %	◯	◯	◯	◯	◯	◯
Squamous aNSCLC Patient %	◯	◯	◯	◯	◯	◯

TREATMENTS

32. What percentage of aNSCLC patients are initially treated based only on their protein-based IHC test for PD-L1?

	0	1% - 25%	26% - 50%	51% - 75%	76% - 99%	100%
Nonsquamous aNSCLC Patient %	◯	◯	◯	◯	◯	◯
Squamous aNSCLC Patient %	◯	◯	◯	◯	◯	◯

33. Among your nonsquamous aNSCLC patients who receive immunotherapy treatment, which biomarker test results do you obtain prior to starting immunotherapy treatment?

- ◯ Actionable genomic alterations only
- ◯ PD-L1 only
- ◯ Both
- ◯ Neither

34. If a patient with aNSCLC (those with nonsquamous and those with squamous) has a treatment plan based on their protein-based IHC test for PD-L1, and the multi-gene panel later identifies a biomarker that would indicate they are a candidate for an FDA approved therapy, do you most often change treatment to the FDA approved therapy in alignment with their genomic biomarker?

- ◯ Yes
- ◯ Yes, but only after treatment which includes a checkpoint inhibitor is either intolerable (high side-effect burden) or ineffective (patient progresses or recurs)
- ◯ No

35. Please indicate your level of agreement with the following statements regarding testing and treatment decisions for your aNSCLC patients.

	Completely disagree	Disagree somewhat	Neither agree nor disagree	Agree somewhat	Completely agree	I don't know
I am always able to get all the biomarker test results I need prior to initiation of first line therapy	◯	◯	◯	◯	◯	◯
If lung tissue is limited, I prioritize the testing for initial histologic diagnosis plus PD-L1 testing, then use blood sample for genomic biomarker testing	◯	◯	◯	◯	◯	◯
It is important to always wait for the biomarkers associated with FDA-approved targeted therapies (i.e., labeled indications) prior to initiation of first line systemic therapy	◯	◯	◯	◯	◯	◯
It is important to test for all actionable biomarkers, including those that have drugs in clinical trials, prior to initiation of first line therapy	◯	◯	◯	◯	◯	◯

36. If a patient with nonsquamous aNSCLC has asymptomatic brain metastases, do you wait to receive all biomarker test results before initiating treatment?

- ◯ Yes
- ◯ No

37. Do you follow one or more guidelines when prescribing treatment options to your patients? [check all that apply]

- ◯ There are no guidelines to which I currently adhere.
- ◯ I follow several guidelines and choose the treatment based on specific clinical scenarios
- ◯ National Comprehensive Cancer Network (NCCN)
- ◯ American Society for Clinical Oncology (ASCO) guidelines
- ◯ Other

37b. Please describe: __________________________________

BARRIERS TO GENOMIC BIOMARKER TESTING

38. When you do NOT order genomic biomarker testing for a patient with either nonsquamous or squamous aNSCLC, how frequently are each of the following factors a reason for NOT ordering?

	Always	Often	Sometimes	Rarely	Never
Patient desire to proceed to immediate treatment initiation without delay for receipt of test results	◯	◯	◯	◯	◯
My own concerns about delaying the patient's treatment while waiting for test results	◯	◯	◯	◯	◯
Lack of insurance coverage for biomarker testing	◯	◯	◯	◯	◯
Need for patient education (e.g., takes too much time, lack of resources [including staff, etc.])	◯	◯	◯	◯	◯
Tumor specimen inadequate for testing (i.e., quantity and/or quality issue)	◯	◯	◯	◯	◯
Uncertainty about which lab/test to use	◯	◯	◯	◯	◯
Concerns that results will not be actionable	◯	◯	◯	◯	◯
Not confident in my ability to make treatment decisions based on results	◯	◯	◯	◯	◯

39. Which of the following factors present challenges to - but do not necessarily prevent - ordering of genomic biomarker testing for your patients with either nonsquamous or squamous aNSCLC?

	Always	Often	Sometimes	Rarely	Never
Patient desire to proceed to immediate treatment initiation without delay for receipt of test results	◯	◯	◯	◯	◯
My own concerns about delaying the patient's treatment while waiting for test results	◯	◯	◯	◯	◯
Lack of insurance coverage for biomarker testing	◯	◯	◯	◯	◯
Need for patient education (e.g., takes too much time, lack of resources [including staff], etc.)	◯	◯	◯	◯	◯
Tumor specimen inadequate for testing (i.e., quantity and/or quality issue)	◯	◯	◯	◯	◯
Uncertainty about which lab/test to use	◯	◯	◯	◯	◯
Concerns that results will not be actionable	◯	◯	◯	◯	◯
Not confident in my ability to make treatment decisions based on results	◯	◯	◯	◯	◯

40. If there are other barriers you encounter when you consider ordering a genomic biomarker test for your patients with aNSCLC, please describe them: ________________________________

STRATEGIES TO FACILITATE GENOMIC AND IMMUNE BIOMARKER TESTING

41. Has your practice implemented any strategies to increase rates of biomarker testing for patients with aNSCLC and any other types of advanced cancer?
◯ Yes
◯ No

42. Please indicate if your strategy(ies) employed any of the following methods:
☐ Clinician education (including pathologist, oncologist, NP, PA, nurse, etc.)
☐ Coordination across multidisciplinary treatment teams (e.g., surgery, radiation therapy, medical oncology, pathology)
☐ Fundraising/philanthropy to assist patients with the cost of testing
☐ Upfront patient cost assessment (i.e., co-pay determination prior to testing)
☐ Proactive outreach to health plans and insurers to ensure coverage (i.e., pre-authorization for insurance coverage)
☐ Enhanced tumor specimen acquisition and quality
☐ Patient education
☐ Practice-wide contract with one or more testing labs
☐ Use of clinical guidelines or pathways
☐ Other [free text]

42b. Please describe: ________________________________

43. Have you evaluated whether your practice's testing rates have improved?
- ◯ Yes, and they have increased
- ◯ Yes, but they have not increased
- ◯ No

43b. Please provide any additional detail of your practice's specific strategy(ies) to increase rates of biomarker testing for patients with advanced stage lung cancer or other types of advanced cancer:

____

44. Are you willing to be contacted about your practice's strategy(ies)?
- ◯ No, You may not contact me
- ◯ Yes, you may contact me, but keep my survey responses anonymous
- ◯ Yes, you may contact me and link my survey responses to my identity

Please provide your name and preferred email address:

Name:

____

Email

____
